# Mechanisms of confluence-dependent expression of CD26 in colon cancer cell lines

**DOI:** 10.1186/1471-2407-11-51

**Published:** 2011-02-01

**Authors:** Masako Abe, Pamela A Havre, Yasuyo Urasaki, Kei Ohnuma, Chikao Morimoto, Long H Dang, Nam H Dang

**Affiliations:** 1 Department of Hematologic Malignancies, Nevada Cancer Institute, Las Vegas, Nevada, 89135, USA; 2 Division of Hematology/Oncology, University of Florida, Gainesville, FL 32610, USA; 3 Division of Clinical Immunology, Advanced Clinical Research Center, Institute of Medical Science, The University of Tokyo, Tokyo, 108-8639, Japan

## Abstract

**Background:**

CD26 (dipeptidyl peptidase IV, DPPIV) is a 110 kDa surface glycoprotein expressed in most normal tissues, and is a potential novel therapeutic target for selected cancers. Our work evaluates the mechanism involved in confluence-dependent CD26 expression in colon cancer.

**Methods:**

Colon adenocarcinoma cells were grown to confluence, and expression of CD26 and transcription factors implicated in its regulation was confirmed by immunofluorescence and Western blotting. Real-time PCR was also performed to evaluate CD26 upregulation at the transcriptional level. The influence of c-Myc on CD26 expression during different growth conditions was further evaluated following transient transfection of a c-Myc-expressing plasmid and a c-Myc specific siRNA.

**Results:**

We found that the colon cancer cell lines HCT-116 and HCT-15 exhibited a confluence-dependent increase in CD26 mRNA and protein, associated with decreased expression of c-Myc, increased USF-1 and Cdx 2 levels, and unchanged HNF-1α expression. Meanwhile, ectopic expression of c-Myc in both cell lines led to decreased CD26 expression. In contrast, transfection of a siRNA targeted to Cdx2 resulted in decreased CD26 level. Importantly, culturing of cells in serum-depleted media, but not acidic conditions, upregulated CD26. While HIF-1α level also increased when cells were cultured in serum-depleted media, its expression was required but not sufficient for CD26 upregulation.

**Conclusions:**

CD26 mRNA and protein levels increase in a confluence-dependent manner in colon carcinoma cell lines, with c-Myc acting as a repressor and Cdx2 acting as an enhancer of CD26 expression. The enhanced expression of CD26 in serum-depleted media and a requirement for HIF-1α suggest a role for nutrients or growth factors in the regulation of CD26 protein expression.

## Background

CD26/dipeptidyl peptidase IV is a 110 kDa surface glycoprotein with diverse functional properties which include peptidase activity and functional and physical association with key molecules in various signal transduction pathways [1,2]. During the past decades, CD26 has been shown to participate in T-cell biology as a costimulatory molecule able to regulate signaling transduction pathways [3-5]. In addition to its role in T-cell biology, recent studies have shown that CD26 also plays an important role in tumor biology.

Furthermore, CD26 itself may be a novel therapeutic target. Anti-CD26 monoclonal antibody (mAb) treatment results in both *in vitro *and *in vivo *antitumor activity against several tumor types, including lymphoma, mesothelioma and renal cell carcinoma [6-8].

However, CD26 expression varies among different cell lines and tumor histologies, resulting in conflicting reports regarding the role of CD26 in the development of different neoplasms, including colon cancer [1]. For example, CD26 is upregulated during enterocytic differentiation of the colon adenocarcinoma cell lines Caco-2 and HT-29 [9]. Interestingly, trypsinizing and seeding late post-confluent Caco-2 cells led to reduced CD26 expression and dipeptidyl peptidase IV activity, as well as the disappearance of other differentiation markers such as sucrase isomaltase and GLUT5 [10]. However, the precise mechanism responsible for this cell density-dependent regulation of CD26 expression and dipeptidyl peptidase IV activity has not been fully elucidated. Compared with most normal tissues, solid tumors tend to exhibit high cell density due to rapid growth, and high cell density has been linked to anti-cancer drug resistance [11,12]. In view of the emerging role of CD26 as a novel therapeutic target, we investigated the mechanism of confluence-dependent expression of CD26 to further understand CD26 biology.

In this paper, we investigated the mechanism involved in the confluence-dependent increase in CD26 expression in the colon cancer cell lines HCT-116 and HCT-15. We demonstrated that c-Myc is involved in the regulation of CD26 expression as a repressor of transcription in both cell lines, and that Cdx2 significantly contributes to the confluence-dependent increase in CD26 expression in HCT-15 cells. Furthermore, we found that serum-depleted culture conditions, but not acidic conditions, significantly upregulated CD26 levels, hence implicating a potential role for nutrients or serum growth factors in the regulation of CD26 protein expression.

## Methods

### Cell culture

The human colorectal carcinoma cell lines HCT-116 and HCT-15 were purchased from the American Type Culture Collection (ATCC, Manassas, VA). HCT 15, HCT-116, and HCT-116 ^HIF1α-/- ^cells [13] were maintained in McCoy's 5A medium (ATCC) supplemented with 10% fetal bovine serum (FBS) (Hyclone, South Logan, Utah), 100 units/ml of penicillin and 100 μg/ml of streptomycin (Invitrogen) at 37°C and 5% CO_2_. For confluence-dependent experiments, HCT-116 cells or HCT-15 cells were seeded at 7.5 × 10^5 ^cells/6-cm dish and 1.0 × 10^6 ^cells/6-cm dish (day-0), respectively, and were grown several days to obtain a post-confluent state. Medium was changed daily. For immunofluorescence staining, cells were grown on a glass slide in RPMI-1640 (Hyclone) without phenol red and supplemented with 10% FBS, 100 units/ml of penicillin, and 100 μg/ml of streptomycin at 37°C. Cells were cultured at sub-confluence or post-confluence by changing medium daily. For normoxic culture conditions, cells were incubated at 37°C, in 5% CO_2 _and room air (21% O_2_). For hypoxic conditions, cells were incubated at 37°C in a hypoxia chamber (Billups-Rothenberg, Del Mar, CA) flushed and equilibrated with 1% O_2_, 94% N_2 _and 5% CO_2 _(Airgas West, Las Vegas, NV). For acidic culture conditions, cells were cultured in McCoy's 5A medium supplemented with 25 mM 2-N-morpholine ethanesulfonic acid (MES, Sigma, St Louis, MO) and 10% FBS (pH 6.2) under normoxic or hypoxic conditions. For hypoxic or acidic culture conditions, cells were used at 3 days post-confluence, and grown on the bottom of a standing flask (25 cm^2^) filled with 50 ml of culture medium to avoid any change in pH or serum concentration. For serum depletion experiments, cells were cultured in McCoy's 5A medium containing 0.3% bovine serum albumin (BSA, Sigma).

### Western blots

Cells were lysed with RIPA buffer [1% NP40, 0.5% deoxycholate, 0.1% SDS, 150 mM sodium chloride, 50 mM Tris-HCl (pH 8.0)] supplemented with Halt protease inhibitor cocktail (Pierce, Rockford, IL), 5 mM EDTA, 5 mM sodium orthovanadate, 10 mM sodium fluoride and 1 mM β-glycerophosphate (Sigma). For preparation of lysates from cells cultured under hypoxic conditions, 100 μM CoCl_2 _was added to the lysis buffer to prevent degradation of HIF-1α. Protein concentration was measured by Micro BCA Protein Assay Kit (Pierce). Lysates were mixed with Laemmli sample buffer (4X), boiled for 5 minutes at 100°C, and equal amounts of protein (50 μg per lane for a 10 comb gel) were separated by SDS-PAGE, followed by overnight wet-transfer to nitrocellulose at 4°C. For detection of CD26, samples were incubated at a lower temperature (37°C for 15 min). CD26 was detected at 220 kDa as a form of homodimer which increased detection sensitivity [14]. We confirmed that the band detected at 220 kDa was depleted in a dose-dependent manner with anti-CD26 mouse monoclonal antibody 1F7 by immunoprecipitation (data not shown). After blocking with 5% nonfat milk in 0.1% Tween 20-TBS for 1 hour at room temperature, membranes were incubated with the appropriate primary antibodies in 1% blocking buffer for 2 hours at room temperature or overnight at 4°C [anti-CD26 (MI1004, Calbiochem, San Diego, CA or AF1180, R&D Systems, Minneapolis, MN), anti-Cdx2 (MU392A-UC, BioGenex, San Ramon, CA), anti-α-tubulin (T9026, Sigma, Saint Louis, MO), anti-c-Myc (9E10), anti-USF-1 (C-20), anti-HNF-1α (C-19) (sc-40, sc-229, and sc-6547, respectively, Santa Cruz, Santa Cruz, CA)], anti-HIF-1α (610958, BD Transduction Laboratories, San Jose, CA)], followed by incubation with the appropriate HRP-conjugated secondary antibodies (Pierce) for 1 hour at room temperature. Membranes were incubated with SuperSignal West Dura (Pierce) for 5 minutes, and visualized using a KODAK Image Station 2000R or 4000R (Carestream Health, New Haven, CT). Some membranes were processed using the Odyssey Infrared Imaging System by using the appropriate infrared-labeled secondary antibodies in Odyssey Blocking Buffer (LI-COR Biosciences, Lincoln, NE).

### Real-time quantitative PCR for CD26 mRNA expression

Total RNA was isolated from cells with RNeasy Mini Kit and QIAshredder (Qiagen, Valencia, CA). Equal amounts of total RNA (90 ng) were analyzed by one step real-time PCR using QuantiFast SYBR Green RT-PCR and Hs_DPP4_1_SG QuantiTect Primer Assay (Qiagen) with the Thermal Cycler 7500 Fast Real Time PCR System (Applied Biosystems, Foster City, CA). The reaction conditions were as follows: 10 minutes at 50°C (RT step), 5 minutes at 95°C, and 40 cycles of 10 seconds at 95°C followed by 30 seconds at 60°C. Relative changes in CD26 mRNA expression were calculated based on the 2^-*ΔCT *^method [15].

### Immunofluorescence microscopy

Following washing with PBS, cells were fixed with cold ethanol for 2 min. Cells were air-dried, rehydrated with PBS, and blocked with 5% BSA in PBS for 30 minutes at room temperature. After washing with PBS for 3 minutes (3 times), cells were incubated with anti-CD26 mouse mAb (202.36, Santa Cruz) for 1 hour at room temperature. Cells were then washed with PBS and incubated with fluorescein isothiocyanate (FITC)-conjugated anti-mouse IgG (F8264, Sigma) for 30 minutes in the dark at room temperature. After washing with PBS, cells were mounted using Fluoroguard™ Antifade (Insitus Biotechnologies, Albuquerque, NM) and immunofluorescence was visualized using a fluorescence microscope (Olympus-BX51, Melville, NY).

### Transient transfection for ectopic expression of c-Myc

Confluent HCT-116 and HCT-15 cells (95-100% confluence) in 6-well plates were transfected with 5 μg/well of pcDNA3-c-Myc (provided by Dr. Wafik El-Deiry, University of Pennsylvania, through Addgene as a distributor, Cambridge, MA) or empty vector using 10 μl/well of Lipofectamine 2000. Cells were grown for several days, and medium was changed daily.

### Transient transfection of Cdx-2 siRNA

HCT-15 cells were grown to 80-90% confluence in 6-well plates and were transfected with 200 pmol/well of Cdx-2 siRNA (sc-43680, Santa Cruz) or control siRNA (sc-37007, Santa Cruz) using 7 μl per well of Lipofectamine 2000 (Invitrogen). Medium was changed daily.

## Results

### Confluence-dependent upregulation of CD26 expression in HCT-116 and HCT-15 cells

**Figure 1:**
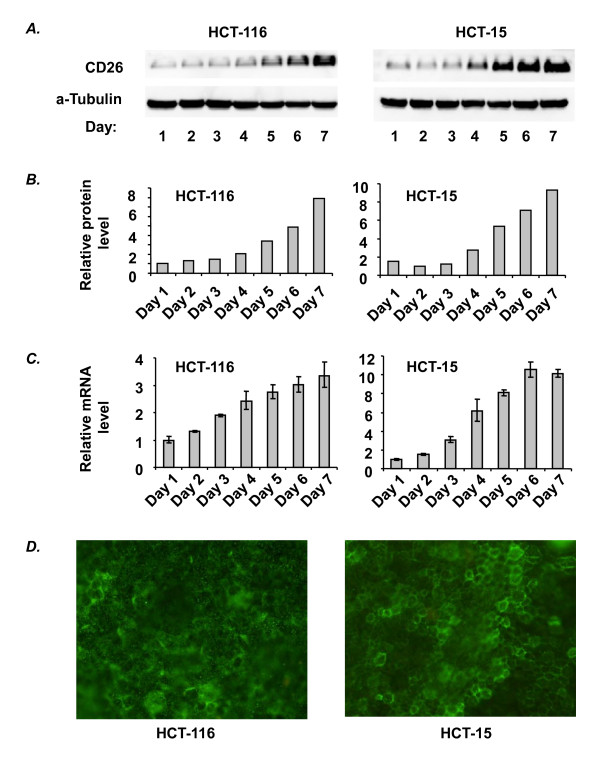
**Confluence-dependent upregulation of CD26 expression in HCT-116 and HCT-15**. A) Confluence-dependent changes in CD26 protein expression were analyzed by Western blots. Cells (1 × 10^6^) were plated in a 6-cm dish (day 0) and grown to post-confluence, with cells being confluent on day 3. Each lane was loaded with 50 μg protein. B) Densitometric analysis of the results from Western blots is shown. C) CD26 mRNA level was measured by real-time quantitative PCR as described in Materials and Methods. D) Immunofluorescence staining of CD26 in HCT-116 and HCT-15 cells on day 7 was performed as described in Materials and Methods. While fluorescence staining was not detectable at sub-confluence (data not shown), membrane localization of CD26 in both cell lines was demonstrated at post-confluence. All data shown are representative of three independent experiments.

Following incubation of the colon cancer cell lines HCT-116 and HCT-15 to a post-confluent state, we detected a confluence-dependent increase in CD26 expression (Figure 1A and 1B), which was regulated at the mRNA level as shown by real-time PCR (Figure 1C). The membrane localization of CD26 in both cell lines at post-confluence was confirmed by immunofluorescence staining (Figure 1D). This confluence-dependent upregulation of CD26 expression was also observed in three other solid tumor cell lines: malignant mesothelioma cell line JMN, colon adenocarcinoma cell line LS174T and gastric cancer cell line MKN45 (data not shown).

### Confluence-dependent expression of transcription factors c-Myc, USF-1, HNF-1α, and Cdx2 in HCT-116 and HCT-15 cells

**Figure 2:**
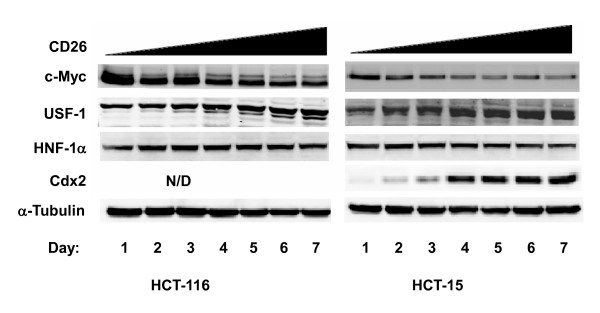
**Confluence-dependent upregulation of transcription factors in HCT-116 and HCT-15 cells**. Culture conditions were the same as for Figure 1. Protein expression was analyzed by Western blotting as described in Materials and Methods. Data shown are representative of three independent experiments. All transcription factors exhibited altered protein expression levels in a confluence-dependent manner except for HNF-1α, which was unchanged. *N/D, Cdx2 was not detected in HCT-116.

To investigate the molecular mechanism involved in the confluence-dependent expression of CD26, we examined the expression of transcription factors known to be involved in regulating CD26 expression. In previous studies, the ubiquitously expressed cellular upstream stimulatory factor 1 (USF-1) and the hepatocyte nuclear factor 1α (HNF-1α) have been reported to exhibit promoter activity for CD26 expression through binding to their consensus motifs in the CD26 promoter region in Caco-2 cells [16,17]. USF-1 is a member of the eukaryotic evolutionarily conserved basic helix-loop-helix leucine zipper transcription factor family [18]. HNF-1α was originally characterized as a liver-specific transcription factor but is also found in other tissues such as kidney, small intestine and thymus [19]. Our data from Western blot analysis show that USF-1 protein expression is increased in a confluence-dependent manner, while HNF-1α expression is unchanged for both cell lines, suggesting that only USF-1 is correlated with the confluence-dependent increase for CD26 expression (Figure 2).

We also examined the expression of the proto-oncogene c-Myc in this process. A transcription factor belonging to the basic helix-loop-helix leucine zipper transcription factor family, c-Myc is closely linked to upregulated proliferation and deregulated cell cycle in tumors [20]. c-Myc is known to bind to specific DNA sequences referred to as E-box sequences. USF-1 can also bind to these sequences, and in fact, c-Myc and USF-1 have been reported to exert opposing effects on promoter activity for some genes by competing with each other to occupy E-box sites [21]. In addition, c-Myc has been reported to suppress gene expression of proteins involved in differentiation and cell cycle arrest such as p15^Ink4b^, p21^Cip1 ^and p27^Kip1^[22]. We now demonstrate that, unlike our findings with USF-1, c-Myc expression decreases in a confluence-dependent manner in both cell lines (Figure 2), suggesting the involvement of c-Myc as a negative regulator of CD26 expression.

Cdx2 is a member of the caudal-related homeobox gene family and has emerged as one of the major regulatory factors controlling intestinal cell differentiation [23]. Previous work has indicated that the forced expression of Cdx2 in HIEC cells (a normal human intestinal epithelial crypt cell model) led to CD26 upregulation [24]. However, Cdx2 can be phosphorylated by cyclin-dependent kinase 2 (Cdk2), resulting in ubiquitin-dependent degradation [25]. Therefore, the increased CD26 expression in HIEC cells was found to be limited by Cdx2 degradation by proteosomes. In our studies, there was a dramatic increase in Cdx2 protein expression in a confluence-dependent manner in HCT-15 cells. However Cdx2 was undetectable in HCT-116 cells at all states of confluence (Figure 2).

### Involvement of c-Myc as a repressor of CD26 expression in HCT-116 and HCT-15 cells

**Figure 3:**
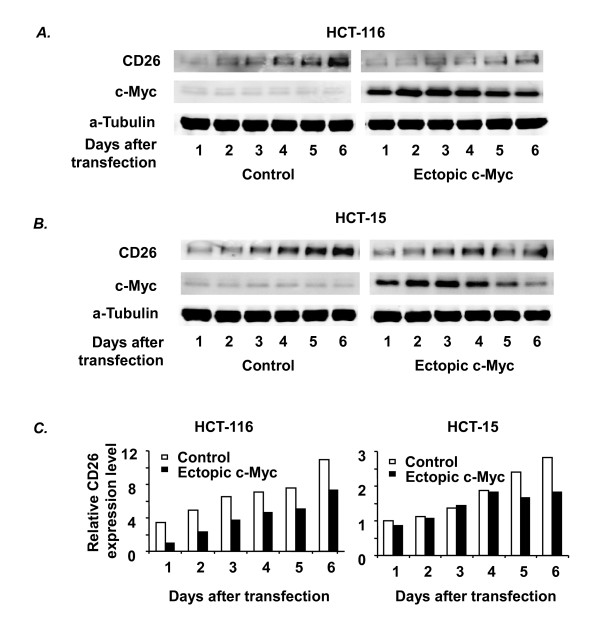
**Effect of c-Myc on confluence-dependent CD26 expression in HCT-116 and HCT-15 cells**. The effect of ectopic expression of c-Myc on the confluence-dependent increase in CD26 protein expression was examined in HCT-116 and HCT-15 cells by transient transfection using either pcDNA3/c-Myc or the empty vector (control) as described in Materials and Methods. Protein expression was analyzed by Western blotting (A, B) and quantified by densitometric analysis (*C*). Data shown are representative of three independent experiments.

To further investigate c-Myc involvement in regulating CD26 expression, we transiently transfected c-Myc into both cell lines at post-confluence. Figure 3 shows that the over-expression of c-Myc resulted in a significant decrease in CD26 protein expression. Although c-Myc over-expression does not completely abrogate the confluence-dependent increase in CD26 expression, these results support a role for c-Myc as a repressor of CD26 expression.

### Involvement of Cdx2 in the confluence-dependent increase in CD26 expression in HCT-15 cells

**Figure 4:**
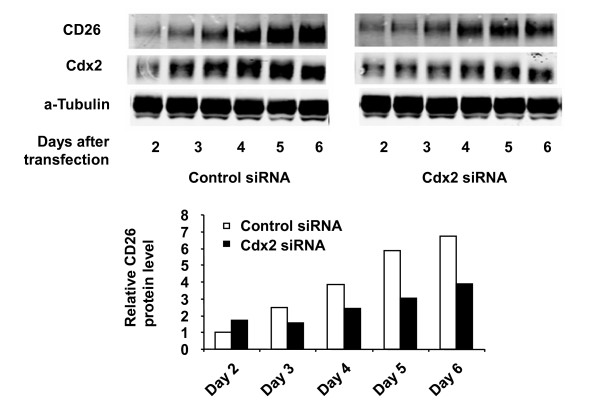
**Effect of Cdx2 siRNA on confluence-dependent CD26 expression in HCT-15 cells**. The effect of Cdx2 siRNA on the confluence-dependent CD26 protein expression in HCT-15 was evaluated following transient transfection (when cells were at 90% confluence) of control siRNA (*left*) or Cdx2 siRNA (*right*) as described in Materials and Methods. CD26 and Cdx2 protein expression were analyzed by Western blotting and quantified by densitometric analysis. Data shown are representative of three independent experiments.

Since we detected a dramatic increase in Cdx2 protein expression along with enhanced CD26 level in a confluence-dependent manner, we next examined whether Cdx2 is involved in the regulation of CD26 expression. As shown in Figure 4, transient transfection of post-confluent HCT-15 cells using Cdx2 siRNA resulted in decreased CD26 expression compared to that seen using the control siRNA. These results indicate a potential role for Cdx2 in regulating the confluence-dependent increase in CD26 expression in this cell line.

To evaluate the effect of ectopic expression of Cdx2 on CD26 level, we transiently transfected Cdx2 into HCT-116 and HCT-15 cells at various states of confluence. However, we found no significant effect on CD26 expression in either cell line (data not shown). One potential explanation for these findings is that Cdx2 may exert its effect on the CD26 promoter by combining with an unknown factor X. Therefore, decreasing Cdx2 level by siRNA would reduce the putative Cdx2-factor X complex, leading to a lowered CD26 promoter activity. This scenario may explain the lack of effect on CD26 transcription following transient transfection of Cdx2 alone (without increasing factor X).

### Effect of hypoxic, acidic and serum-depleted culture conditions on CD26 expression in HCT-116 cells

**Figure 5:**
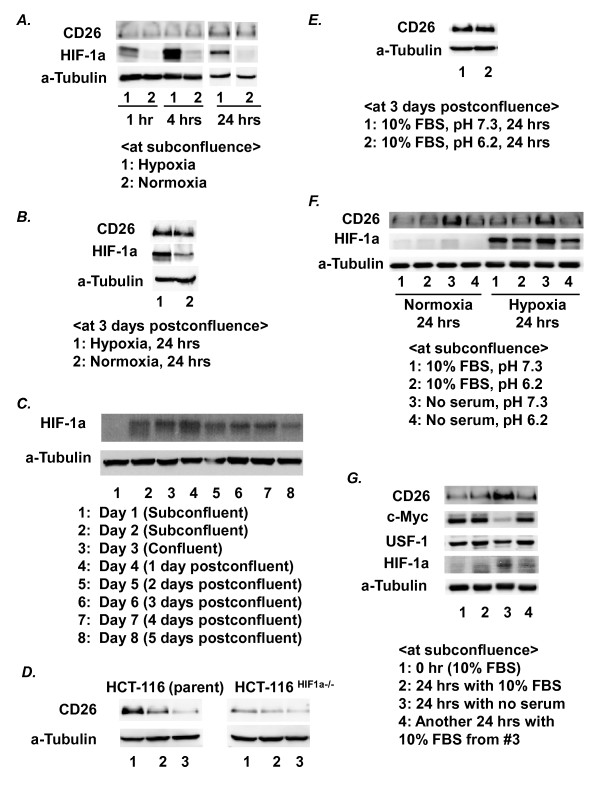
**Effect of hypoxic, acidic and serum-depleted culture conditions on CD26 expression in HCT-116 cells**. A) Effect of hypoxia (1% O_2_) on CD26 protein expression in cells at sub-confluence. Cells at ~50% confluence were incubated under hypoxic or normoxic conditions. Cells were harvested at different time points and protein expression was analyzed by Western blotting as described in Materials and Methods. B) Effect of hypoxia on CD26 protein expression at 3 days post-confluence. Cells were grown to 3 days post-confluence, then incubated under hypoxic or normoxic conditions for 24 hours. C) Expression of HIF-1α at sub- and postconfluence. Cells were grown for 8 days, from sub-confluence through 5 days post-confluence and protein expression was analyzed by Western blotting. D) Enhanced CD26 expression in parental HCT-116 cells compared to HCT-116^HIF1-α-/- ^cells. Whole cell lysates (24 μg) were run on gels and analyzed as described previously. Cells were held at confluence for 3 days (lane 1), 1 day (lane 2), or were sub-confluent (lane 3). E) The effect of acidic conditions on CD26 expression. Cells were grown to 3 days post-confluence as described above, and then incubated in regular 10% FBS medium (pH 7.3) or in acidic medium supplemented with 25 mM MES (pH 6.2) for 24 hours. F) The effect of hypoxia, acidic medium, and serum depletion was examined in cells at sub-confluence. *G*) The effect of switching cells from serum-depleted culture conditions to 10% FBS medium was evaluated. Cells were grown to ~25% confluence (lane #1) and then cultured in regular 10% FBS medium (lane #2) or serum-depleted medium (lane #3) for 24 hours. Cells cultured in serum-free medium for 24 hours were re-cultured in regular 10% FBS medium for another 24 hours (lane# 4). Cell confluence at this point was still ~90%. All data discussed above are representative of at least three independent experiments.

To determine cell culture conditions that might have an effect on the regulation of CD26 expression, we first evaluated the effect of hypoxia. The upregulation of CD26 expression under hypoxic conditions has been reported previously by ourselves and others [26-28], as well as the existence of pericellular hypoxia in dense cultures [12]. Sub-confluent HCT 116 cells were incubated either under normoxic or hypoxic conditions (see Materials and Methods) for 1, 4, or 24 hours. After a 24 hour incubation, cells were still sub-confluent (~90%), and there was no difference in pH between normoxic and hypoxic media. Cells were harvested at the specified time, lysed, run on gels, and analyzed by Western blots (Figure 5A). CD26 expression did not change under hypoxic conditions even when expression of HIF-1α was at its highest (following 4 hr hypoxia). In addition, there was no difference between normoxic and hypoxic cells with respect to CD26 at 3 days post-confluence (Figure 5B). Although hypoxia alone did not lead to the enhancement of CD26 (Figure 5A and 5B), HIF-1α level was increased as cells became more confluent (Figure 5C). It was not detected on day 1 (sub-confluent), but rose steadily until it peaked at day 4 (1 day post-confluent), then declined gradually through day 8 (5 days post-confluent). Comparison of CD26 expression in confluent and sub-confluent HCT-116 and HCT-116 ^HIF1α-/- ^cells revealed significantly higher CD26 expression for cells that are HIF-1α positive (Figure 5D). These results suggest that HIF-1α is required, but not sufficient, for CD26 upregulation.

We next examined whether low extracellular pH affected CD26 protein expression. HCT-116 cells at post-confluence or sub-confluence were cultured in regular 10% FBS medium at pH 7.3 or acidic 10% FBS medium supplemented with 25 mM MES at pH 6.2. As shown in Figure 5E and 5F, the pH of the medium did not affect CD26 expression in sub-confluent or post-confluent cultures.

Since nutrients and serum growth factors may be depleted when cells reach a high cell density, we also tested the effect of culturing cells in serum-depleted media. Since the supply of available nutrients and growth factors may vary in post-confluent cultures, the use of serum-depleted media may offer a more consistent culture environment to evaluate the role of nutrients and serum growth factors in CD26 expression. When HCT-116 cells at sub-confluence were cultured in 0.3% BSA medium without serum for 24 hours, CD26 protein level was significantly increased compared to cells cultured in 10% FBS medium (Figure 5F and 5G). HIF-1α level was likewise increased when cells were cultured in the absence of serum (Figure 5G). On the other hand, c-Myc expression was decreased in serum-depleted media. Interestingly, CD26 expression in cells cultured for 24 hours with 10% FBS (following 24-hour serum depletion) returned to basal level as did HIF1-α and c-Myc (Figure 5G), demonstrating the reversibility of this phenomena.

## Discussion

In the present study, we demonstrate that CD26 expression is increased in colon carcinoma lines grown to confluence, associated with changes in selected transcription factors. In particular, we found that c-Myc expression decreases with confluence, and that it can act as a repressor of CD26 expression. In contrast, Cdx2 expression parallels that of CD26, increasing with confluence. A potential explanation for the observed confluence-dependent increase in Cdx2 protein expression may be due to confluence-mediated cell cycle arrest, resulting in decreased expression of Cdk2 and decreased phosphorylation of Cdx2 by Cdk2, leading to increased Cdx2 protein expression [25]. Our findings also suggest the existence of a co-factor X that has a role in regulating CD26 expression in combination with Cdx2, although additional studies will need to be done to test this hypothesis. In addition, we demonstrated that a hypoxia-induced increase in HIF-1α was not associated with an enhanced CD26 level, but that HIF-1α expressing cells displayed a higher level of HIF-1α along with a higher level of CD26 when grown to confluence. In comparison, a lack of enhancement of HIF-1α and CD26 levels in HCT-116 ^HIF1α-/- ^cells, suggest that HIF-1α expression is required but not sufficient for increased expression of CD26.

The growth of tumors is known to be associated with hypoxia and low extracellular pH in the tumor microenvironment [29]. In our experimental system, low extracellular pH had no effect on CD26 protein expression, regardless of the extent of cell confluence. On the other hand, we found that depletion of serum factors was associated with a robust upregulation of CD26. Furthermore, the effect of serum depletion on CD26 protein expression was reversible, as was also the case with c-Myc and HIF-1α expression. All three proteins returned to basal level of expression in 24 hours following culturing in regular 10% FBS medium. These results hence suggest that there is a serum factor(s)-dependent regulatory mechanism for CD26 protein expression, which affects cells maintained without serum at sub-confluence. Since the half life of CD26 protein in cells at post-confluence is reported to be much longer than 48 hours as assayed by [^35^S]-methionine label pulse-chase experiments [9], our results suggest the existence of an unidentified serum factor-dependent degradative mechanism in the regulation of CD26 protein expression. These data indicate that a post-confluent state can be mimicked by cells at sub-confluence by depleting required serum factors. While we did not detect differences in CD26 expression when cells were incubated in serum-free conditions with low levels of BSA (data not shown), it is still theoretically possible that the presence of BSA in the culture media of cells cultured in serum-free conditions can interfere with CD26 metabolism and expression. We would also like to note that our observations are restricted to the specific cells and proteins evaluated in our experimental conditions, and are not necessarily applicable to other cell types or proteins. When cells are at post-confluence, depletion of nutrients is likely to limit cell growth. In addition, tight junctions between cells growing at post-confluence may inhibit binding of serum factors to the cell surface, leading to the upregulation of CD26. However, the potential contribution of factors secreted from cells at post-confluence cannot be excluded. In fact, others have reported increased HIF-1α expression when sub-confluent breast carcinoma cells were incubated with conditioned media from confluent cultures [12].

Our previous studies demonstrated the potential of targeted therapy using anti-CD26 mAb treatment in several tumor models, including renal clear cell carcinoma and malignant mesothelioma [7,8]. In these cell lines, upregulation of p27^Kip1 ^was observed following treatment with anti-CD26 mAb. We observed that p27^Kip1 ^expression was elevated in cells treated with anti-CD26 mAb compared to isotype-matched control mAb when treatment was done in serum-depleted culture conditions (data not shown). However, this anti-CD26 mAb-mediated enhancement in p27^Kip1 ^level was not observed if cells were incubated in serum-containing media, indicating that serum depletion can enhance the effect of anti-CD26 mAb treatment in cells normally expressing low CD26 level (presumably by upregulating target expression). Future studies will examine in detail the mechanism associated with CD26 enhancement mediated by serum-depleted culture conditions. This knowledge, combined with our present findings, may prove to have clinical significance by expanding the potential usage of anti-CD26 mAb therapy in selected human cancers through enhanced CD26 expression.

## Conclusions

In summary, we demonstrate that CD26 expression is regulated in a confluence-dependent manner, involving potentially transcriptional and posttranslational mechanisms. For the confluence-dependent increase in CD26 expression, decreased expression of c-Myc and increased expression of USF-1 and Cdx2 may contribute to the upregulation of CD26 expression at the transcriptional level. Meanwhile, factors associated with serum depletion may also contribute to increased CD26 expression, with one potential mechanism being at the posttranslational level by regulating an unknown serum factor-dependent CD26 protein degradation pathway. Importantly, our present findings may result in the expansion of the use of CD26-targeted therapy in the clinical setting for selected tumors by regulating the expression of CD26 itself.

## Competing interests

The authors declare that they have no competing interests.

## Authors' contributions

MA carried out the experiments. PAH, YU, KO, CM, LHD, and NHD participated in the design of the study, its coordination, and helped to draft the manuscript. In addition, LHD was responsible for the creation of the HCT-116 ^HIF1α-/- ^cell line.

All authors read and approved the final manuscript.

## Pre-publication history

The pre-publication history for this paper can be accessed here:

http://www.biomedcentral.com/1471-2407/11/51/prepub
